# Effects of Nicorandil on Inflammation, Apoptosis and Atherosclerotic Plaque Progression

**DOI:** 10.3390/biomedicines9020120

**Published:** 2021-01-27

**Authors:** Max Lenz, Christoph Kaun, Konstantin A. Krychtiuk, Patrick Haider, Mira Brekalo, Nadine Maier, Laura Goederle, Christoph J. Binder, Kurt Huber, Christian Hengstenberg, Johann Wojta, Philipp J. Hohensinner, Walter S. Speidl

**Affiliations:** 1 Department of Internal Medicine II—Division of Cardiology, Medical University of Vienna, 1090 Vienna, Austria; max.lenz@meduniwien.ac.at (M.L.); christoph.kaun@meduniwien.ac.at (C.K.); konstantin.krychtiuk@meduniwien.ac.at (K.A.K.); patrick.haider@meduniwien.ac.at (P.H.); mira.brekalo@meduniwien.ac.at (M.B.); nadine.maier@meduniwien.ac.at (N.M.); christian.hengstenberg@meduniwien.ac.at (C.H.); johann.wojta@meduniwien.ac.at (J.W.); walter.speidl@meduniwien.ac.at (W.S.S.); 2 Ludwig Boltzmann Institute for Cardiovascular Research, 1090 Vienna, Austria; 3 Department of Laboratory Medicine, Medical University of Vienna, 1090 Vienna, Austria; laura.goederle@meduniwien.ac.at (L.G.); christoph.binder@meduniwien.ac.at (C.J.B.); 4 Research Center for Molecular Medicine of the Austrian Academy of Sciences, 1090 Vienna, Austria; 5 3rd Medical Department for Cardiology and Emergency Medicine, Wilhelminenhospital and Sigmund Freud University, 1160 Vienna, Austria; Kurt.huber@meduniwien.ac.at; 6 Core Facility Imaging, Medical University of Vienna, 1090 Vienna, Austria

**Keywords:** nicorandil, inflammation, apoptosis, atherosclerosis, plaque, plaque stabilization

## Abstract

Nicorandil, a balanced vasodilator, is used in the second-line therapy of angina pectoris. In this study, we aimed to illuminate the effects of nicorandil on inflammation, apoptosis, and atherosclerotic plaque progression. Twenty-five LDL-R -/- mice were fed a high-fat diet for 14 weeks. After 6 weeks mice were randomly allocated to treatment with nicorandil (10 mg/kg/day) or tap water. Nicorandil treatment led to a more stable plaque phenotype, displaying an increased thickness of the fibrous cap (*p* = 0.014), a significant reduction in cholesterol clefts (*p* = 0.045), and enhanced smooth muscle cell content (*p* = 0.009). In endothelial cells nicorandil did not reduce the induction of adhesion molecules or proinflammatory cytokines. In H_2_O_2_ challenged endothelial cells, pretreatment with nicorandil significantly reduced the percentage of late apoptotic/necrotic cells (*p* = 0.016) and the ratio of apoptotic to living cells (*p* = 0.036). Atherosclerotic lesions of animals treated with nicorandil exhibited a significantly decreased content of cleaved caspase-3 (*p* = 0.034), lower numbers of apoptotic nuclei (*p* = 0.040), and reduced 8-oxogunanine staining (*p* = 0.039), demonstrating a stabilizing effect of nicorandil in established atherosclerotic lesions. We suggest that nicorandil has a positive effect on atherosclerotic plaque stabilization by reducing apoptosis.

## 1. Introduction

Cardiovascular diseases (CVD) are the leading cause of death within the western world. According to the Cardiovascular Disease Statistics 2019 published by the European Society of Cardiology (ESC), a heterogeneous development of CVD-associated risk factors was reported. Besides a decline in smoking, hypertension, and alcohol consumption, an increased prevalence of obesity and diabetes mellitus was highlighted. Among ESC member countries, 3.6 million new cases of coronary artery disease (CAD) were recorded during the year 2017. In terms of mortality, CAD made up approximately 40% of all CVD related deaths [1]. Similar findings were indicated in the Heart Disease and Stroke Statistics 2020 published by the American Heart Association (AHA). In 2017, CAD caused 365,914 deaths in the US, accounting for about 13% of total mortality [2].

A cardinal symptom of CAD is angina pectoris. The etiology of angina has been described heterogeneously; however, accompanying symptoms are caused by a mismatch in myocardial oxygen supply and demand. In most patients, this imbalance is a direct result of flow-limiting stenoses within the coronary arteries [3]. Although stable CAD is associated with a proportionally low mortality rate, inflammation within coronary plaques and activation of the coagulation system causes plaque disruption and thrombus-formation, leading to acute coronary events [4].

In addition to revascularization strategies in patients with large myocardial areas at risk, different pharmacological approaches are recommended in the treatment of stable CAD. First-line therapy with anti-ischemic drugs includes short-acting nitrates (immediate relief of effort angina), beta-blockers (BB), and calcium channel blockers (CCB). Despite the lack of randomized controlled trials (RCT), CCB and BB are regarded as the first choice over second-line anti-ischemic drugs as a primary therapeutic strategy. Indicated second-line treatment currently consists of the drugs nicorandil, ranolazine, ivabradine, and trimetazidine [5].

Nicorandil, a balanced vasodilator, is known to exert anti-ischemic properties via dual mechanisms of action. As an NO-donor, it stimulates guanylyl cyclase, increasing intracellular cyclic guanosine monophosphate (cGMP) levels. Furthermore, nicorandil acts as an activator of ATP-dependent potassium channels (kATP), causing cellular hyperpolarization. Both mechanisms contribute to the relaxation of vascular smooth muscle cells, resulting in a decrease of cardiac pre-load as well as after-load and an improved coronary blood flow [6]. In an RCT with 5126 patients, nicorandil was demonstrated to improve the primary composite endpoint (death due to CAD, non-fatal myocardial infarction, and unplanned hospital admission for cardiac chest pain) compared to placebo treatment. Additionally, nicorandil reduced the rate of acute coronary syndromes and the rate of all cardiovascular events [7].

For nicorandil, evidence of antiatherogenic properties in a mouse model and modulation of plaque composition towards a more stable phenotype in humans were described [8]. Moreover, a previous study suggested anti-inflammatory effects via TLR4/MyD88/NF-κB signaling [9]. Interestingly, one of the related side effects of nicorandil is ulcer formation of the gastrointestinal and urogenital tract. Several case reports indicate partly severe manifestations with signs of acute/chronic inflammation [10,11,12]. In addition, nicorandil was demonstrated to exhibit antiapoptotic effects in various cell types [13,14,15,16]. The mechanism of action behind these findings is believed to be mediated through activation of mitochondrial kATP channels, resulting in an attenuation of the mitochondrial apoptotic pathway [14,16,17].

A significant body of evidence describes atherosclerosis as a chronic inflammatory process affecting the vessel wall. Accumulation and peroxidation of LDL particles trigger a robust inflammatory response, causing the expression of adhesion molecules and leading to the migration of immune cells [18]. Besides inflammation, apoptosis was found to play a pivotal role in the development of atherosclerotic lesions. Depending on the observed cell type, a multitude of factors contribute to the described progression. Endothelial cell death is possibly involved in the increased production of cytokines and growth factors, further enhancing plaque formation. Moreover, endothelial damage promotes a procoagulatory status, resulting in thrombogenesis [19]. Regarding smooth muscle cells (SMC), apoptosis leads to a decreased collagen synthesis and therefore a thinning of the fibrous cap. Consequently, this causes reduced plaque stability and a phenotype prone to rupture [20]. In early apoptotic stages, SMCs were reported to contribute to amplified thrombogenicity due to raised thrombin generation [19]. The effects of apoptosis on macrophages seem to differ depending on the stage of plaque development as well as the phagocytic capacity of the cells. In advanced stages, clearance of apoptotic remnants may result in a protracted inflammatory activation and enlarged necrotic cores. In summary, apoptotic cell death of various cell types seems to effectuate atherosclerotic plaque progression and might contribute to plaque destabilization [21]. Therefore, antiapoptotic drug-effects could lead to plaque stabilization and reduce cardiovascular events.

This study aimed to elucidate whether nicorandil has direct effects on atherosclerotic plaque progression. Therefore, we investigated the effect of nicorandil in an LDL-R -/- model with already preexisting atherosclerotic lesions rather than evaluating the impact of nicorandil on de novo atherogenesis. Furthermore, we were interested in a possible effect of nicorandil on inflammation and apoptosis in activated endothelial cells.

## 2. Experimental Section

### 2.1. Mouse Treatment

For this study, 25 LDL-receptor knockout (LDL-R -/-) mice on a C57/BL6 background were used (schematic overview found in Supplemental Figure S1). The animals were bred and held in the animal facility of the Medical University of Vienna. Housing conditions were in line with the latest guidelines on the ethical use of laboratory animals. A 12 h/12 h light/dark cycle was established, with a fixed room temperature of 22 °C and air humidity ranging from 50–60%. Conventional chow diet and water ad libitum were provided until the age of 12 weeks. Consecutively, a high-fat diet (HFD; 21% butterfat, Ssniff, Soest, Germany) was implemented. This approach was chosen to establish an atherosclerotic plaque before starting drug treatment and granting insight into the atherosclerotic progression rather than into the impact on de novo atherogenesis. At the age of 18 weeks, the mice were randomly allocated to a treatment (*n* = 12) or a control group (*n* = 13). The treatment group received 10 mg/kg/day nicorandil via tap water, whereas mice in the control group received tap water only. According to Reagan-Shaw et al., the drug dose was calculated from the maximum human daily dose using weight and body surface (40 mg/day) [22]. A similar dose was already used in a previous study [8]. At the age of 26 weeks, mice were sacrificed to analyze plaque extent and composition. All animals were male littermates. Experiments were carried out in accordance with recommendations of the ethics committee of the Medical University of Vienna (No. GZ: 66.009/0172-WF/V/3b/2016). The three R’s (replacement, reduction, and refinement) of ethics in animal testing were followed and taken into account while planning the experiments.

### 2.2. Atherosclerotic Plaque Staining

The extent of the atherosclerotic plaque was determined by en face staining. After harvesting the whole aorta and cleaning and pinning it to a paraffin plate, Sudan IV staining (Merck, Darmstadt, Germany) was used to visualize the plaque-covered area. The final analysis was carried out by a blinded member of the study team using ImageJ software (V1.8.0, Bethesda, MD, USA). The preparation of the aortic root sinus was done according to an established protocol [23]. Masson’s trichrome staining in the aortic root was utilized to determine plaque extent, fibrous cap thickness, collagen content, and cholesterol clefts. Macrophage and smooth muscle cell infiltration of the lesion were analyzed using anti-CD68 (Abcam, Cambridge, UK) and anti-alpha smooth muscle actin antibody (anti-α-Sm-1; Abcam, Cambridge, UK) according to the manufacturer’s instructions. Cleaved caspase-3 was stained using a suitable antibody (Thermo Fisher Scientific, Waltham, MA, USA). Apoptotic nuclei were made visible utilizing TUNEL Assay (Promega, Madison, WI, USA). For indirect staining of reactive oxygen species (ROS) via oxidative DNA damage, anti-8-Oxoguanine antibody (ab206461, Abcam, Cambridge, UK) was used according to the manufacturer’s instructions following prior digestion using pepsin (Sigma-Aldrich, Waltham, MA, USA) [24]. Starting with the appearance of all three aortic valve leaflets, 50 µm atherosclerotic plaque was analyzed (10 µm between each section). All tissue sections were analyzed using TissueFAXS (V4, TissueGnostics, Vienna, Austria) as well as ImageJ software. All analyses were carried out by a blinded member of the study team.

### 2.3. Endothelial Cell Culture

Human umbilical vein endothelial cells (HUVEC) were isolated from fresh umbilical cords and cultured thereafter [25]. If not otherwise indicated, cells were pretreated with 500 µM nicorandil (Merck, Darmstadt, Germany) for 30 min, followed by stimulation with 200 U/mL Interleukin-1 beta (IL-1ß, Merck, Darmstadt, Germany). For all experiments, six different pools of HUVEC were used, each one consisting of cells from at least three independent donors. All human material was obtained and processed according to the hospital’s ethics committee and security board’s recommendations.

### 2.4. Static Adhesion Assay

Adhesion under static conditions was determined as described previously [25]. In short, peripheral venous blood was taken from healthy donors. CD66b positive selection kit (Stemcell Technologies, Grenoble, France) was used according to the manufacturers’ instructions to isolate polymorphonuclear leukocytes (PMN) from whole blood. A confluent HUVEC monolayer was pretreated with 500 µM nicorandil (Merck, Darmstadt, Germany) for 30 min, followed by stimulation with 200 U/mL IL-1ß (Merck, Darmstadt, Germany). After an incubation period of 4 h, cells were washed three times with a medium (M199, Sigma-Aldrich, St. Louis, MI, USA). Subsequently, 1 mL of medium containing 1 × 10^6^ PMN was added to the wells and incubated for 30 more minutes. Afterward, wells were washed three times with phosphate buffered saline (PBS). A Zeiss Axiovert 40 CFL light microscope with a 10× lens was used to examine cell adhesion. The average number of adherent granulocytes per picture section (10 per well) was determined by images acquired with a Zeiss Axiocam ICc3 camera. The analysis was carried out by a blinded member of the study team using ImageJ software.

### 2.5. Flow Cytometry

Flow cytometry was performed using a FACS Canto II system (Becton Dickinson, Franklin Lakes, NJ, USA). HUVEC were pretreated with 5 µM–500 µM nicorandil (Merck, Darmstadt, Germany) for 30 min, followed by stimulation with 200 U/mL IL-1ß (Merck, Darmstadt, Germany). After an incubation period of 4 h, cells were washed with cold PBS (4 °C), scratched, and transferred to suitable tubes. Cells were stained for the expression of E-selectin (PE, CD62E, BD Pharmingen, San Diego, CA, USA), VCAM-1 (PE-Cy5TM CD106, BD Pharmingen, San Diego, CA, USA), and ICAM-1 (FITC, CD54, Beckman Coulter, Carlsbad, CA, USA). Mean fluorescence intensity was analyzed by FACS Diva software (BD). For staining of late apoptotic/necrotic cells, HUVEC were pretreated with 500 µM nicorandil for 30 min. Subsequently, 25 µM H2O2 was added for a duration of 4 h. A Dead Cell Apoptosis Kit (Thermo Fisher Scientific, Waltham, MA, USA) containing Annexin V (PE) and 7-AAD Viability Staining Solution (Biolegend, San Diego, CA, USA) was used according to manufacturers’ instructions for staining the cells. An Attune NxT Flow Cytometer and the associated Attune NxT Flow Cytometer Software (both Thermo Fisher Scientific, Waltham, MA, USA) were used to record and interpret data.

### 2.6. mRNA Purification and Quantitative PCR

HUVEC were pretreated with 500 µM nicorandil (Merck, Darmstadt, Germany) for 30 min, followed by stimulation with 200 U/mL IL-1ß (Merck, Darmstadt, Germany) for 2 h. Consecutively, RNA was isolated using a High Pure RNA Isolation Kit (Roche, Mannheim, Germany). For cDNA, reverse transcription was performed using a Transcriptor First Strand cDNA Synthesis Kit (Roche, Mannheim, Germany). Quantitative PCR was performed using a LightCyclerTaqMan Master (Roche, Mannheim, Germany) system according to manufacturers’ instructions. Primers were designed using the Roche Universal ProbeLibrary: E-selectin (forward primer: 5′ accagcccaggttgaatg-3′, reverse primer: 5′-ggttggacaaggctgtgc-3′, UPLprobe #86; Amplicon Size [bp] 89), VCAM-1 (forward primer: 5′ tgaatctaggaaattggaaaaagg-3′, reverse primer: 5′- tgaatctctggatccttaggaaa –3′, UPLprobe #39; Amplicon Size [bp] 69), ICAM-1 (forward primer: 5′-ccttcctcaccgtgtactgg-3′, reverse primer: 5′-agcgtagggtaaggttcttgc-3′, UPLprobe #71; Amplicon Size [bp] 90), and MCP-1 (forward primer: 5′-ttctgtgcctgctgctcat-3′, reverse primer: 5′-ggggcattgattgcatct-3′, UPLprobe #83; Amplicon Size [bp] 90). GAPDH (forward primer: 5′-agccacatcgctcagacac-3′, reverse primer: 5′-gcccaat-acgaccaaatcc-3′, UPLprobe #60; Amplicon Size [bp] 66) was used as a housekeeping gene. Amplification conditions consisted of an initial incubation at 95 °C for 10 min, followed by 45 cycles of 95 °C for 10 s, 63 °C for 20 s, and 72 °C for 6 s, followed by a final cooling phase of 40 °C. LightCycler Software Version 3.5 (Roche, Mannheim, Germany) was utilized to perform data analysis.

### 2.7. Protein Determination

HUVEC were pretreated with 500 µM nicorandil (Merck, Darmstadt, Germany) for 30 min, followed by stimulation with 200 U/mL IL-1ß (Merck, Darmstadt, Germany). After 8 h, supernatants were collected from the cell culture wells. ELISA for interleukin-6 (IL6; R & D systems, Minneapolis, MN, USA) and interleukin-8 (IL8; Thermo Fisher Scientific, Waltham, MA, USA) were used according to the manufacturer’s instructions to measure levels of IL6 and IL8.

### 2.8. Statistical Analysis

All data are presented as mean ± SD. When analyzing two conditions, the Mann-Whitney U test was used for nonparametric data (determined by the Kolmogorov-Smirnov test), whereas the student’s t-test was used for parametric data. Multiple comparison analysis was achieved by ANOVA, followed by Bonferroni correction. Values of *p* < 0.05 (two-tailed) were considered statistically significant. For all statistical analyses, SPSS Statistics (V26, IBM, Armonk, NY, USA) was utilized.

## 3. Results

### 3.1. Effects of Nicorandil on Plaque Size and Stability in LDL-R -/- Knockout Mice

**Figure 1:**
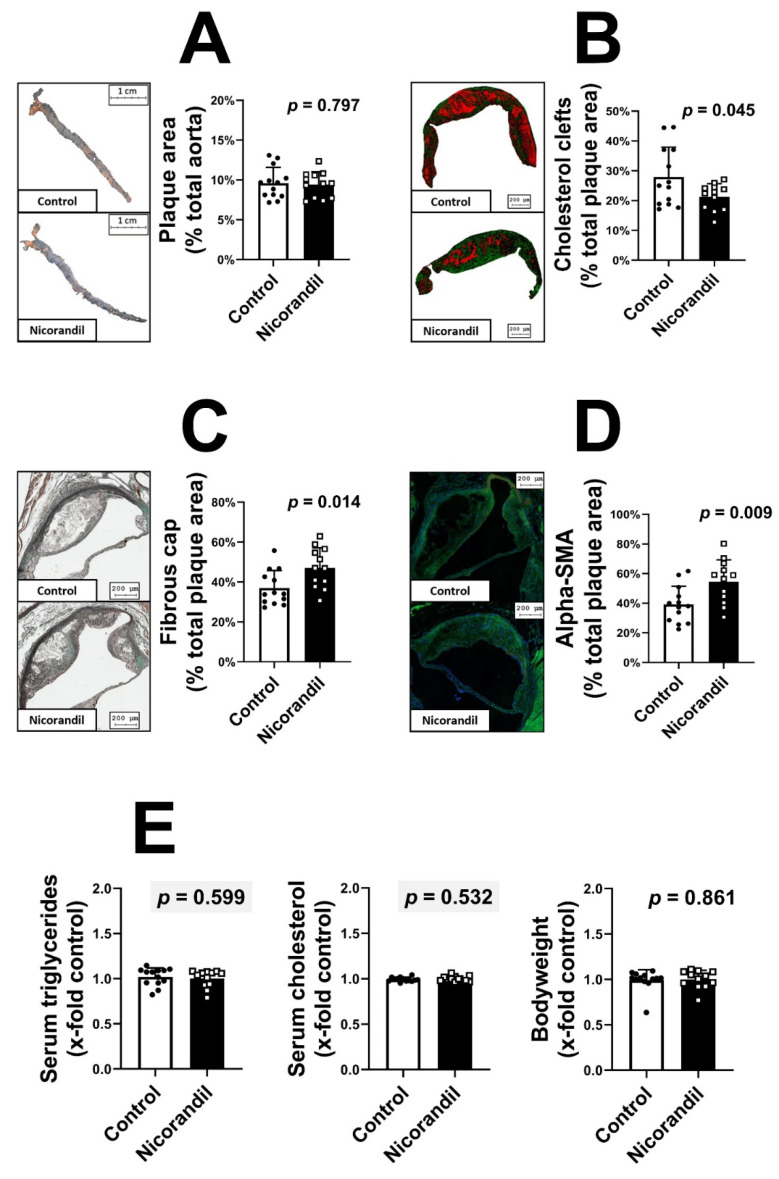
Atherosclerotic plaque development after nicorandil treatment. Atherosclerotic plaque formation was induced by a high-fat diet in 25 LDL-R -/- mice. The animals were randomized to either a control group (*n* = 13) or a nicorandil treatment group (*n* = 12, 10 mg/kg/day). No difference in plaque size was observable between the groups, as determined by en face staining (**A**). Nicorandil treatment led to a significant reduction in cholesterol clefts. Images show software analysis of TissueFAXS scans of Mason Trichrome staining in a false color format (**B**). In addition, nicorandil treatment led to a significant increased thickness of the fibrous cap (**C**) and an increased smooth muscle cell content (**D**). No difference in serum triglyceride levels, cholesterol levels, and body weight (**E**) was observed at time of sacrifice. All values given represent means ± standard deviations; the statistical significance of *p* ≤ 0.05 was considered significant.

To understand the effect of nicorandil treatment on atherosclerotic disease progression, we used 25 LDL-R -/- mice (schematic overview found in Supplementary Figure S1). A high-fat diet was implemented six weeks before starting the treatment with nicorandil (10 mg/kg/day). This approach was deliberately chosen to observe the potential effects on atherosclerotic progression rather than on de novo atherogenesis. At the age of 26 weeks, all animals were sacrificed, and plaque extent and composition were analyzed. En face staining of the aorta was performed to evaluate plaque burden. Nicorandil treatment showed no effect on overall plaque formation (*p* = 0.797, Figure 1A). Furthermore, no changes were observable within the aortic root (nicorandil: 17.04% ± 6.27% vs. control: 19.71 ± 5.82%, *p* = 0.505). When analyzing the atherosclerotic lesion composition, a significant reduction in cholesterol clefts was found in nicorandil treated animals (*p* = 0.045, Figure 1B). Furthermore, an increased thickness of the fibrous cap area was detected (*p* = 0.014, Figure 1C), suggesting increased plaque stability. This held true, as absolute thickness was found to be increased (nicorandil: 124.12 μm ± 30.14 μm vs. control: 98.99 μm ± 18.85 μm, *p* = 0.019) in addition to the percentage area of the plaque. Smooth muscle cell content was measured by staining of anti-α-Sm-1. Treatment with nicorandil significantly increased smooth muscle cell content compared to the control group (*p* = 0.009, Figure 1D). To determine the inflammatory status of the lesion, macrophage infiltration was quantified by staining CD68+ cells. Nicorandil treatment had no observable effect on the atherosclerotic plaque infiltration (data not shown, *p* = 0.832). No difference in serum triglyceride levels (*p* = 0.599, Figure 1E), cholesterol levels (*p* = 0.532, Figure 1E), and body weight (*p* = 0.861, Figure 1E) was observed comparing control and nicorandil treated animals.

### 3.2. Effects of Nicorandil on Endothelial Adhesion Molecules

**Figure 2:**
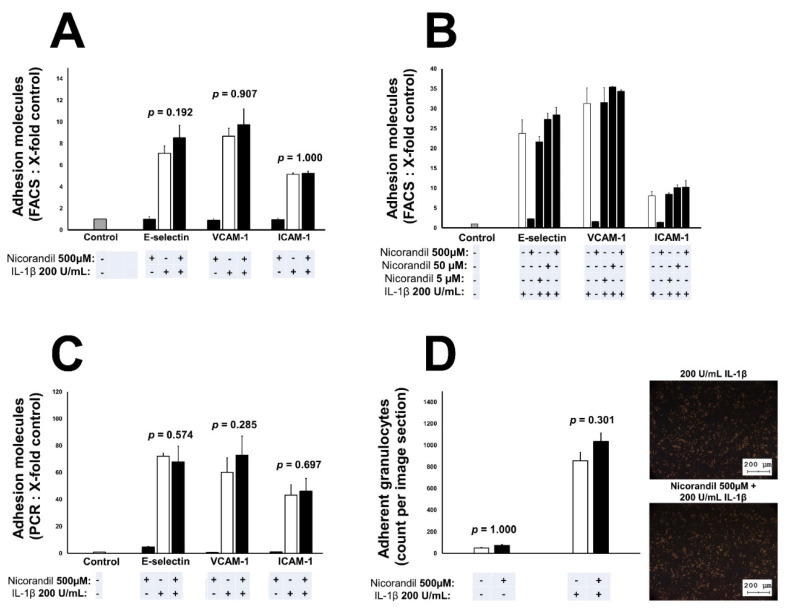
Effect of nicorandil treatment on adhesion molecules in endothelial cells. In endothelial cells, treatment with nicorandil (5 μM–500 μM) led to no difference in the expression of adhesion molecules 4 h after IL-1β stimulation (determined by flow cytometry, (**A**) and (**B**)). Similar findings were made for mRNA levels of E-selectin, VCAM-1, and ICAM-1, as they remained unchanged in endothelial cells 2 h after IL-1β stimulation (**C**). Granulocytes adhesion did not differ in nicorandil pretreated endothelial cells compared to vehicle control 4 h after IL-1β stimulation (**D**). All values given represent means ± standard deviations; the statistical significance of *p* ≤ 0.05 was considered significant.

Regarding the controversial available data on the potential pro-/anti-inflammatory properties of nicorandil, we used an in vitro cell culture approach to evaluate the expression of inflammatory induced endothelial adhesion molecules. Treatment with nicorandil had no effect on IL-1β induced E-selectin (*p* = 0.192), VCAM-1 (*p* = 0.907), and ICAM-1 (*p* = 1.00) surface expression determined by flow cytometry (Figure 2A). Furthermore, the expression of adhesion molecules remained unchanged independent of the used nicorandil concentration, ranging from 5 µM to 500 µM (Figure 2B). We were able to confirm FACS data using real-time quantitative PCR. Nicorandil treatment had no impact on E-selectin (*p* = 0.574), VCAM-1 (*p* = 0.285), and ICAM-1 (*p* = 0.697) mRNA levels 2 h after stimulation with IL-1ß (Figure 2C). As a functional readout, granulocytes adhesion to a nicorandil pretreated endothelial monolayer was examined. Within the static adhesion assay, no difference between nicorandil and vehicle control was observable (*p* = 0.301, Figure 2D). The used concentration of nicorandil was deemed non-toxic to endothelial cells as determined by lactate dehydrogenase (LDH) cytotoxicity assay (data not shown, *p* = 0.993).

### 3.3. Effects of Nicorandil on Inflammation Associated Interleukins and MCP-1

**Figure 3:**
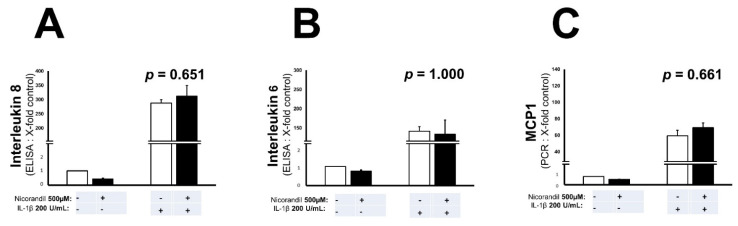
Pro-inflammatory cytokine production after nicorandil treatment. No significant reduction of IL8 (**A**) and IL6 (**B**) protein levels were observable in nicorandil (500 μM) treated endothelial cells 8 h after stimulation with IL-1β. Furthermore, there was no change in MCP-1 mRNA levels 2 h after stimulation with IL-1β, as determined by real-time PCR (**C**). All values given represent means ± standard deviations; the statistical significance of *p* ≤ 0.05 was considered significant.

To further illuminate a potential modulation of inflammation, IL8 and IL6 levels were measured in cell culture supernatant 8 h after stimulation with IL-1ß. Protein content was determined by ELISA. Nicorandil treatment had no effect on the expression of IL8 (*p* = 0.651, Figure 3A) as well as IL6 (*p* = 1.000, Figure 3B) levels. Additionally, as determined by real-time quantitative PCR, no changes in MCP-1 mRNA levels were detectable following nicorandil treatment (*p* = 0.661, Figure 3C).

### 3.4. Effects of Nicorandil on Apoptosis In Vitro

**Figure 4:**
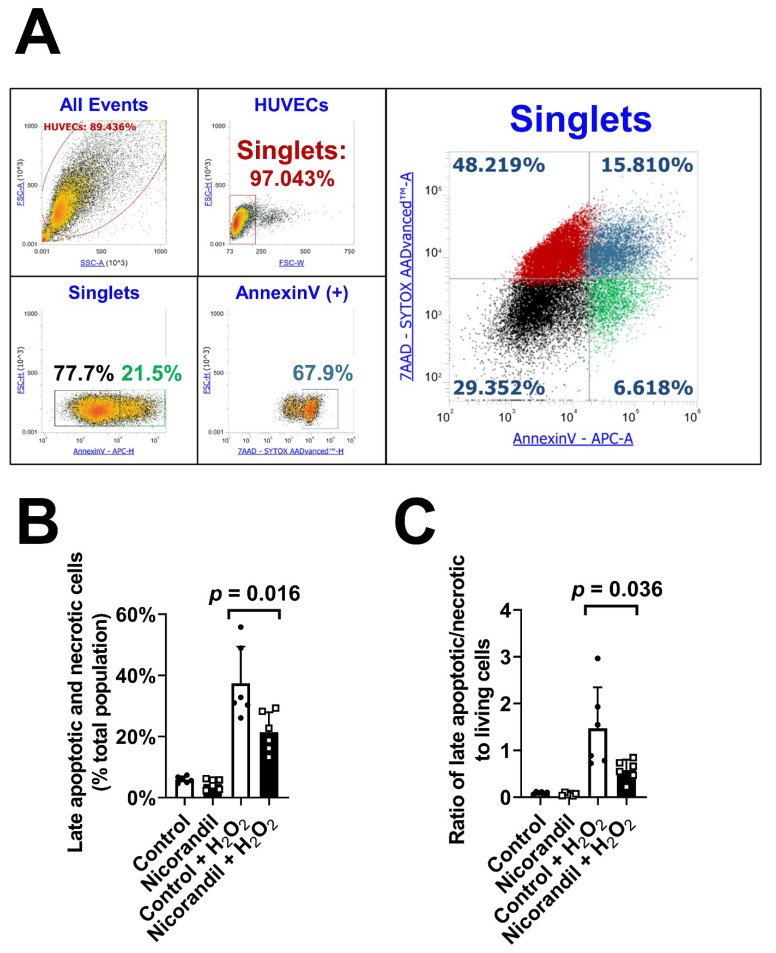
Effect of nicorandil treatment on apoptosis in endothelial cells. (**A**) Gating strategy; cell debris was excluded from analysis using forward/sideward scatter plot. Subsequently, singlets were gated using FSC width vs. FSC height and split into AnnexinV +/- and 7-AAD +/- cells. Backgating into the singlet population revealed double-positive (late apoptotic/necrotic) and double-negative cells (intact, living cells). A total of 25 µM H_2_O_2_ was added to pretreated endothelial cells (500 µM nicorandil) for a duration of 4 h. Pretreatment with nicorandil significantly reduced the percentage of late apoptotic/necrotic cells (**B**) and decreased the ratio of late apoptotic/necrotic to living cells (**C**). All values given represent means ± standard deviations; the statistical significance of *p* ≤ 0.05 was considered significant.

To determine if previously described antiapoptotic effects would be relevant in endothelial cells, nicorandil was used in a cell culture approach. H_2_O_2_ challenged cells were stained with Annexin V and 7-AAD and consecutively analyzed using flow cytometry (gating strategy shown in Figure 4A). Pretreatment with nicorandil significantly reduced the percentage of late apoptotic/necrotic cells (*p* = 0.016, Figure 4B). Moreover, it led to a decreased ratio of late apoptotic/necrotic to living cells (*p* = 0.036, Figure 4C).

### 3.5. Effects of Nicorandil on Apoptosis In Vivo

**Figure 5:**
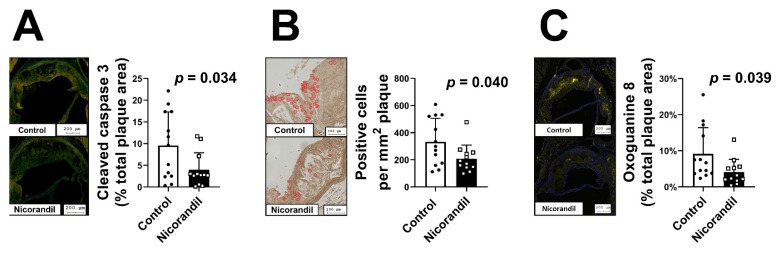
Effect of nicorandil treatment on apoptosis in vivo. Atherosclerotic plaque formation was induced by a high-fat diet in 25 LDL-R -/- mice. The animals were randomized to either a control group (*n* = 13) or a nicorandil treatment group (*n* = 12, 10 mg/kg/day). Nicorandil treatment led to a significantly decreased cleaved caspase-3 content (**A**), a significantly lower number of apoptotic nuclei (**B**), and decreased expression of oxidative DNA damage (**C**). All values given represent means ± standard deviations; the statistical significance of *p* ≤ 0.05 was considered significant.

To confirm in vitro indications for reduced apoptosis, we followed on the antiapoptotic findings in vivo. Staining of cleaved caspase-3 and TUNEL assay were performed on aortic root sections of LDL-R -/- mice with and without nicorandil treatment. Cleaved caspase-3 content was significantly decreased in animals treated with nicorandil as compared to the control group (*p* = 0.034, Figure 5A). Furthermore, the number of apoptotic nuclei was significantly lower in animals receiving nicorandil (*p* = 0.040, Figure 5B). Indirect ROS staining was achieved by quantifying oxidative DNA damage. An anti-8-Oxoguanine antibody was utilized to highlight plaque areas heavily affected by oxidative damage. Nicorandil-treated animals displayed a significantly decreased expression within the analyzed atherosclerotic lesions (*p* = 0.039, Figure 5C).

## 4. Discussion

We were able to demonstrate a plaque-stabilizing effect of nicorandil treatment during atherosclerotic plaque progression. Although there was no observable difference in total plaque size, nicorandil treated LDL-R -/- mice displayed an increased fibrous cap thickness, paired with decreased necrotic core formation (indicated by cholesterol cleft formation) and enhanced infiltration of smooth muscle cells. Our in vitro results suggest that the mode of action of the beneficial effects of nicorandil is via its antiapoptotic properties.

A possible effect of nicorandil on atherosclerotic plaques was already reported previously. In an atherosclerotic mouse model, nicorandil was described as leading to a reduction of atherosclerotic plaque formation [8]. However, the high-fat diet and nicorandil treatment were started simultaneously in the described mouse model, leading to a de novo atherogenic rather than an atherosclerotic model, and thus is not comparable to the situation in humans. We therefore decided to use a model where the atherosclerotic lesion was already established before starting drug treatment. This approach translates more closely to the actual human situation, since nicorandil is used for symptom relief in patients with angina, that is, when atherosclerotic stenosis already causes symptoms. Our findings are reflected by human data previously published, as human subjects treated with nicorandil displayed no difference in plaque size, while the percentage of fibrous cap tissue was reported to be significantly increased [8]. Additionally, the percentage of cholesterol cleft formation was found to be considerably decreased, pointing towards a more stable plaque formation. Taking previous work and our data into account, it is assumable that nicorandil treatment has no direct effect on the growth/progression of already established atherosclerotic plaques. However, we present evidence for a positive modulation towards a more stable plaque phenotype. In an RCT with more than 5000 patients, nicorandil improved the primary composite endpoint, led to a reduced rate of acute coronary syndromes, and lowered the overall rate of cardiovascular events [7]. These effects might be attributed to the described plaque-stabilizing effect of nicorandil.

As atherosclerosis is known to be a chronic inflammatory process, we aimed to elucidate the potential pro-/anti-inflammatory effects of nicorandil. Studies found in the literature paint a diverse picture: on the one hand, there are reports of anti-inflammatory properties; on the other hand, many case reports outline partly severe ulcer formation with signs of acute/chronic inflammation [10,11,12,26,27,28,29,30,31]. Since the focus of our study was on CAD and atherosclerotic plaque composition, we chose a cell culture approach utilizing endothelial cells. In the described setup, nicorandil did not affect any measured markers of inflammation. There was no observable change in the expression of adhesion molecules determined by flow cytometry and real-time PCR. As there could be a dose-dependent effect, nicorandil was tested in concentrations ranging from 5 µM to 500 µM, producing the same results. Additionally, inflammation-associated cytokines were measured within the supernatant of endothelial cells. Nicorandil pretreatment did not affect MCP-1, IL8, and IL6 protein levels. Moreover, macrophage infiltration in our in vivo atherosclerosis model of the atherosclerotic lesion remained unaltered after treatment. In conclusion, our data propose that nicorandil does not exert a direct anti- or pro-inflammatory effect.

Since there is evidence suggesting the antiapoptotic effect of nicorandil, we aimed to illuminate these findings further using an endothelial cell culture setup. The mentioned effects were already demonstrated in various cell lines [13,14,15,16,32,33,34]. Further, a pivotal connection of apoptosis in vascular smooth muscle cells and plaque stability had already been demonstrated [20]. Nicorandil’s underlying antiapoptotic mechanism of action was described to be mediated through the activation of mitochondrial kATP channels [14,16,17]. A mitochondrial kATP-channel antagonist was shown to abolish these antiapoptotic properties, further strengthening these claims [34]. Following nicorandil induced kATP-channel opening, antiapoptotic effects are considered to be induced via the PI3K/Akt pathway [34,35]. In line with this, caspase-3, which is involved in the final steps of the PI3K/Akt pathway, was found to be reduced after treatment with nicorandil [36,37]. We were able to confirm the mentioned antiapoptotic properties in our study. After pretreatment with nicorandil, H_2_O_2_-challenged cells displayed a significantly reduced percentage of late apoptotic/necrotic cells.

Furthermore, our findings indicate a decreased ratio of late apoptotic/necrotic cells to living cells. To affirm the effects in vivo, we performed staining for cleaved caspase-3 and DNA damage in aortic root sections. LDL-R -/- mice treated with nicorandil exhibited significantly less cleaved caspase-3 within the atherosclerotic lesion. Moreover, the number of apoptotic nuclei was found to be substantially lower compared to the control group. Therefore, we suggest that the lesion stabilizing effect of nicorandil is due to its antiapoptotic properties. Major differences in apoptotic cells were found within the outer layers of the atherosclerotic plaque, indicating at least a contribution of antiapoptotic effects in endothelial cells to the plaque stabilizing phenotype.

Although nicorandil did not exert any pro- or anti-inflammatory effects in our in vitro setup, a reduction in ROS-associated DNA damage was found within the atherosclerotic lesions of nicorandil-treated animals. This might be a direct effect of nicorandil, as suggested previously [38]. However, this finding might also be caused by the reduction of apoptotic remnants, which would lead to a protracted inflammatory activation. [21]. Hence a reduced overall cellular stress due to nicorandil treatment within the atherosclerotic lesion might indirectly reduce inflammation. This could explain the previously suggested anti-inflammatory properties of nicorandil in vivo, as apoptotic cells might fuel the inflammatory cascade [8,9].

## 5. Conclusions

We would like to highlight three significant findings of this work. We were able to demonstrate a stabilizing effect of nicorandil in established atherosclerotic lesions. Our data propose no anti-/pro-inflammatory properties of nicorandil in vitro, regardless of the concentration used. We were able to confirm the propagated antiapoptotic effect of nicorandil in our in vitro and in vivo setups. Additionally, we are the first to link the influence of nicorandil on increasing atherosclerotic plaque stability to a reduction of apoptosis within the atherosclerotic lesion in vivo.

## Data Availability

Upon reasonable request data supporting the results can be obtained from the corresponding author.

## Supplementary Materials

The following are available online at https://www.mdpi.com/2227-9059/9/2/120/s1, Figure S1: Graphical overview of mouse experiment.
