# Supplementary figures and images for: Effects of Nicorandil on Inflammation, Apoptosis and Atherosclerotic Plaque Progression
Source: Biomedicines. 2021 Jan 27;9(2):120. doi: 10.3390/biomedicines9020120 (PMC7912627; doi:10.3390/biomedicines9020120)

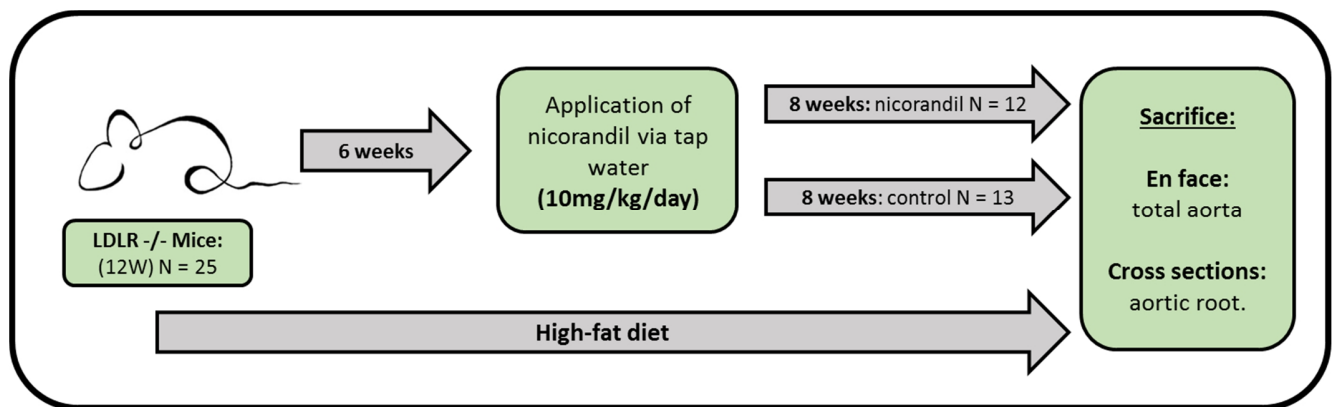

Supplementary Figure 1: Overview of the treatment regime.

Supplement: Supplementary file 1 [file biomedicines-09-00120-s001.pdf]
